# Immunogenicity and safety of quadrivalent versus trivalent inactivated influenza vaccine: a randomized, controlled trial in adults

**DOI:** 10.1186/1471-2334-13-224

**Published:** 2013-05-20

**Authors:** Jiří Beran, Mathieu Peeters, Walthère Dewé, Jolana Raupachová, Lenka Hobzová, Jeanne-Marie Devaster

**Affiliations:** 1 The Vaccination and Travel Medicine Center, Poliklinika II, Bratří Štefanu 895, Hradec Králové 500 03, Czech Republic; 2 Department of Infectious Diseases, University Hospital, Hradec Králové, Czech Republic; 3 GlaxoSmithKline Vaccines, Rixensart, Belgium

**Keywords:** Adjuvant, Influenza vaccine, Tetravalent, Trivalent inactivated, Quadrivalent

## Abstract

**Background:**

Two phylogenetic lineages of influenza B virus coexist and circulate in the human population (B/Yamagata and B/Victoria) but only one B-strain is included in each seasonal vaccine. Mismatch regularly occurs between the recommended and circulating B-strain. Inclusion of both lineages in vaccines may offer better protection against influenza.

**Methods:**

This study (NCT00714285) assessed the immunogenicity and safety of two candidate quadrivalent influenza vaccines (QIV) containing two A- and two B-strains (one from each lineage) in adults (18–60 years). Subjects were randomized and stratified by age to receive either QIV (non-adjuvanted or low-dose adjuvanted [LD QIV-AS]) or trivalent influenza vaccine (TIV, non-adjuvanted or low-dose adjuvanted [LD TIV-AS]), N = 105 in all treatment groups. The study evaluated the statistical non-inferiority of the immunological response elicited by QIV and LD QIV-AS versus TIV and LD TIV-AS and the statistical superiority of the response elicited by the quadrivalent vaccines against the B-strain (B/Jiangsu) not included in the TIV.

**Results:**

Pre-defined non-inferiority and superiority criteria were reached for both QIVs compared to the TIVs. On Day 21 in all vaccine groups SCRs were ≥54.8%, SPRs ≥88.5% and SCFs ≥5.4 for the A strains and B strain included in all vaccines (B/Malaysia). This fulfilled the European (CHMP) and the US (CBER) licensing criteria for the assessment of influenza vaccines in adults (CHMP criteria: SCR > 40%, SPR > 70%, SCF > 2; CBER criteria: LL of 95% CI for SPR ≥ 70% or SCR ≥ 40%). Only the QIVs met the CHMP and CBER criteria for the B/Jiangsu strain. In the QIV and LD-QIV-AS groups, the SCFs were 9.1 and 8.1, respectively and the SPRs were 98.1% and 95.2%, whereas for the TIV and LD-TIV-AS groups, the SCFs were 2.3 and 2.5, respectively, and the SPRs were 75.0% and 63.8%, with the LLs of the 95% CI <70% for SPR and <40% for SCR.

**Conclusions:**

Addition of a fourth strain did not impact the immune response elicited by the three original strains contained in the TIV. A clear immunological benefit was seen with the QIV formulation for the second B-strain, indicating that quadrivalent vaccines could provide broader protection against influenza.

**Trial registration:**

ClinicalTrials.gov: NCT00714285

## Background

Seasonal influenza affects 5 to 15% of the worldwide population in all age groups every year, and causes high morbidity and mortality, as well as substantial socio-economic disruption [1]. Currently, the influenza viruses in circulation in the human population are A(H1N1), A(H3N2) and two distinct lineages of B-strain: the B/Victoria/2/87 and B/Yamagata/16/88 lineages [2]. These have coexisted since 1983 in the human population [3], and both strains have circulated broadly worldwide since 1988 [1,2,4,5].

The B influenza strain is the major cause of an epidemic every 2 to 4 years, with infection occurring in all age groups [6]. Influenza B causes a substantial number of hospitalizations and deaths and, although seasonal mortality estimates are highest for the H3N2 strain, the impact of B-strains is greater than that of H1N1 [7,8]. From 1976 to 1999, 16% of the influenza-associated deaths in the US were caused by influenza B and occurred mostly in the elderly population, although they also represent 46% of all influenza related death in children below the age of 5 years [8].

Current seasonal influenza vaccines include the two A-strains (H1N1 and H3N2) but only one B-strain [9]. The prediction for which B-strain to include in the vaccine is not always accurate [6], giving rise to a variable level of mismatch between the B-strain contained in the vaccine and the B-strain causing infection and disease each season. In 6 of the past 11 years, in the US, the main circulating B-virus did not match the strain recommended and included in the vaccine [10]. In the EU, mismatch occurred in 4 out of 8 seasons between the 2003/2004 and 2010/2011 Northern Hemisphere influenza seasons [11]. During seasons with a mismatch of B-strains, protection is reduced in adults and even more so in children [12-15] because cross-reactivity between the two B-strain lineages is limited [2,16,17]. In vaccine-naïve children, live attenuated influenza vaccines have a high vaccine efficacy against well-matched B-viruses, but provide only poor or no cross-protection against strains of the opposite B-lineage [13]. In order to ensure increased vaccine coverage against viruses of both B-lineages, one option may be to include one strain of each lineage in the influenza vaccine, and in doing so potentially offer a better protection against influenza disease [16,18]. In recognition of the value of a quadrivalent influenza vaccine, the World Health Organization for the first time in February 2012 issued a recommendation for a strain of the alternate B lineage to be included in quadrivalent influenza vaccines made for the 2012–2013 Northern Hemisphere immunization campaigns [19].

Adjuvanted vaccines have been shown to be highly immunogenic and well tolerated in children and adults, providing an important antigen-sparing strategy [20,21] and cross immunogenicity between clades of the same lineage [21]. Thus, the use of adjuvants may be a way to address possible concerns regarding the manufacturing capability to handle a fourth strain within the short timeframe of seasonal production.

This study was the first conducted by GlaxoSmithKline Vaccines to assess, in adults aged 18 to 60 years, the immunogenicity and safety of an influenza vaccine containing four strains (two A-strains: H1N1 and H3N2 and two B-strains: Victoria and Yamagata lineages), with and without adjuvant, and using 2 different doses of hemagglutinin (15 μg and 5 μg hemagglutinin in the unadjuvanted and adjuvanted formulations, respectively, for each strain).

## Methods

### Study design

The study was approved by the study center ethics committee and conducted in accordance with Good Clinical Practice (GCP) and all applicable regulatory requirements including the Declaration of Helsinki. Written informed consent was obtained from all participants prior to study entry. This was a Phase I/II, single-center, single-blind, controlled study conducted in the Czech Republic (registered at http://www.ClinicalTrials.gov: NCT00714285). The study ran from July 2008 until January 2009, with approximately 6 months duration per subject. Participants were randomized on a 1:1:1:1 basis to four parallel treatment groups using an internet-based system that employed a minimization procedure accounting for center and age. Each participant received one intramuscular injection, into the deltoid region of the non-dominant arm, of their assigned vaccine: quadrivalent influenza vaccine (QIV), low-dose adjuvanted QIV (LD QIV-AS), trivalent influenza vaccine (TIV), or low-dose adjuvanted TIV (LD TIV-AS). The participants were unaware of the treatment assigned during the course of the trial. Blood samples were collected prior to immunization (Day 0) and 21 days post-vaccination (Day 21). Vaccine administration during study was recorded in an electronic case report form. A summary of the protocol is available at http://www.gsk-clinicalstudyregister.com (ID 111295).

### Study population

Participants were healthy adults aged 18 to 60 years who the investigator believed would comply with the study requirements. Women of childbearing age were required to use reliable contraception. Exclusion criteria included: pregnant or lactating women, confirmed influenza infection during the previous year, influenza vaccination during the 2007–2008 season, a history of hypersensitivity to an influenza vaccine or any component of the vaccine, any immunosuppressive or immunodeficient condition.

### Vaccines

The vaccines used were all inactive split-virion vaccines manufactured by GlaxoSmithKline (GSK) Vaccines. All vaccines contained the A/Solomon Islands/03/2006 [H1N1] IVR-145 strain, A/Wisconsin/67/2005 [H3N2] NYMCX strain and B/Malaysia/2506/2004 strain (Victoria lineage), according to recommendations for the 2007–2008 season [22]. The QIV vaccines included an additional strain: the B/Jiangsu/10/2003 strain (Yamagata lineage). The QIV and TIV vaccines contained 15 μg hemagglutinin (HA) of each strain, whereas the adjuvanted formulations (LD QIV-AS and LD TIV-AS) contained 5 μg HA of each strain. Both adjuvanted vaccines contained 62.5 μL of AS03, an α-tocopherol, oil-in-water emulsion-based Adjuvant System (squalene 10.69 mg, tocopherol 5.8 mg). All vaccines were supplied in pre-filled syringes containing a 0.5 mL dose.

### Study objectives

The first primary objective was to evaluate, in terms of hemagglutination inhibition (HI) , the statistical non-inferiority of the immunological response elicited by the quadrivalent vaccines compared to the trivalent vaccines (QIV vs. TIV and LD QIV-AS vs. LD TIV-AS) for the three recommended seasonal strains. A co-primary objective aimed at assessing the statistical superiority of the immunological response elicited by the quadrivalent vaccines compared to the trivalent vaccines (QIV vs. TIV and LD QIV-AS vs. LD TIV-AS) for the B/Jiangsu strain, not included in the TIV formulations. Secondary objectives were to evaluate the statistical non-inferiority of the immunological response measured by HI and elicited by LD QIV-AS vs. QIV, and to assess the safety and reactogenicity of the four study vaccines in terms of solicited local and general adverse events (AEs) 7 days post-vaccination, unsolicited AEs during the 21 days post-vaccination and serious adverse events (SAEs) and occurrence of potential immune-mediated diseases (pIMD) during the 6-month study period.

### Immunogenicity evaluation

All serum samples were analyzed in the GlaxoSmithKline Vaccines laboratories: HI assay was performed in GlaxoSmithKline laboratory located in Laval, Canada and neutralization assay in Dresden, Germany. HI was assessed by a validated HI micro-titer assay using chicken erythrocytes against the four vaccine strains [23]. Geometric mean titers (GMTs), seroconversion rates (SCRs), seroprotection rates (SPRs) and seroconversion factors (SCFs) were calculated with their 95% confidence intervals (CI). SCR was defined as the percentage of subjects with either a pre-vaccination HI titer <10 and a post-vaccination titer ≥40 or a pre-vaccination titer ≥10 and a minimum four-fold increase in post-vaccination titer. SPR was defined as the percentage of subjects with a serum HI titer ≥40. SCF was defined as the fold increase in serum HI GMT per strain on Day 21 compared to Day 0. Assessment of the HI response was based on the CHMP and the US Food and Drug Administration (FDA) Center for Biologics Evaluation and Research (CBER) guidance targets for seasonal influenza vaccines [24,25]. The CHMP criteria for subjects aged 18 to 60 years are that the point estimate of SPR is >70%, or SCR is >40% or SCF is >2. The CBER criteria for subjects aged 18 to 64 years are that the lower limit of the 95% CI for SPR is ≥70% or for SCR is ≥40%.

Microneutralizing (MN) antibody titers against both B-strains were also assessed according to previously described methods [26,27]. The 50% neutralization titers were calculated using the Reed and Muench method [28]. The cut-off was a MN titer of 1:28 with participants considered to be seronegative if no neutralizing activity was present at a base dilution of 1:28. Seronegative samples were assigned a titer of 14 for calculations. As no protective neutralizing correlate is established, GMT and SCR (defined as ≥4-fold increase in titer relative to the baseline value) were used to characterize the immune response.

### Reactogenicity and safety evaluation

All participants were provided with diary cards to record solicited and unsolicited AEs. Local (pain, redness and swelling at the injection site) and systemic (arthralgia, fatigue, headache, myalgia, nausea, shivering and fever) AEs were recorded on the day of vaccination and for the six subsequent days.

### Statistical analysis

The target sample size was 100 participants per group with the assumption that approximately 5% of participants would not be evaluable for immunogenicity endpoints. Thus, 420 subjects were planned to be enrolled into this study with 105 per group. This sample size was not driven by a power calculation, but assuming a standard deviation of 0.5 for the individual HI titers, a precision of 16% was expected on the GMT ratio estimates of interest. Demographic characteristics were tabulated as a whole and per vaccine group. The analysis of the HI response was based on the per-protocol cohort for immunogenicity. GMTs, SCRs, SCFs and SPRs were all estimated with their 95% CIs.

Non-inferiority and superiority objectives were assessed for each vaccine strain by estimating GMT ratios (QIV / TIV) 21 days after vaccination. GMTs were estimated using an Analysis of Covariance (ANCOVA) model including treatment as the fixed effect and baseline as covariate was fitted on log_10_ transformed post-vaccination HI titer for each strain. Adjusted GMT ratios were obtained from these models with their 95% CIs. In terms of GMT ratio, non-inferiority and superiority were demonstrated if the lower bound of the 95% CIs of the ratio estimates were higher than 0.67 and 1, respectively. The MN response was analyzed in a subset of participants belonging to the per-protocol cohort for immunogenicity. Both GMTs and SCRs were estimated for each group for only the B-strains. The analysis of safety was performed on the total vaccinated cohort (TVC). Incidence rates of AEs with 95% CIs were calculated for each group.

## Results

### Study population

**Figure 1:**
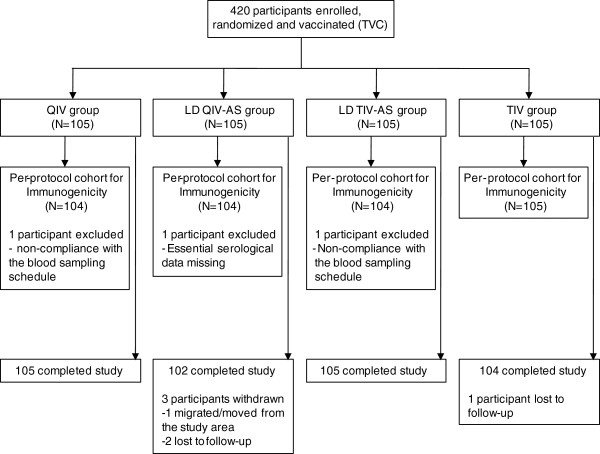
**Participant flow diagram.** Abbreviations: N = number of participants; LD QIV-AS = low-dose of quadrivalent influenza vaccine adjuvanted with AS03; LD TIV-AS = low-dose of trivalent influenza vaccine adjuvanted with AS03; QIV = quadrivalent influenza vaccine; TIV = trivalent influenza vaccine; TVC = total vaccinated cohort No participants were withdrawn due to SAEs.

In July 2008, 420 participants (105 in each treatment group) were enrolled and vaccinated. Overall, 416 subjects completed the study: 105 in the QIV group, 104 in the TIV group, 102 in the LD QIV-AS group and 105 in the LD TIV-AS group (Figure 1). All but one participant were of White-Caucasian/European heritage. The mean age was 37.6 years and 60.0% were female (Table 1).

**Table 1:** Baseline demographics of the per-protocol cohort for immunogenicity

**Treatment group**^**a**^					
**Characteristic**	**QIV**	**LD QIV-AS**	**LD TIV-AS**	**TIV**	**Total**
N^b^	104	104	104	105	417
Age (years)					
Mean ± SD	38.6 ± 11.82	37.7 ± 12.30	36.7 ± 12.84	37.4 ± 12.51	37.6 ± 12.35
Median	38.0	37.0	33.5	36.0	36.0
Range (min–max)	19–59	18–59	18–59	18–59	18–59
Gender n(%)					
Female	60 (57.7)	66 (63.5)	66 (63.5)	58 (55.2)	250 (60.0)
Male	44 (42.3)	38 (36.5)	38 (36.5)	47 (44.8)	167 (40.0)
Race n(%)					
African heritage	1 (1.0)	0	0	0	1 (0.2)
White Caucasian	103 (99.0)	104 (100)	104 (100)	105 (100)	416 (99.8)

^a^ Groups were administered the quadrivalent influenza vaccine (QIV), the low-dose adjuvanted quadrivalent influenza vaccine (LD QIV-AS), the low-dose adjuvanted trivalent influenza vaccine (LD TIV-AS) or the trivalent influenza vaccine (TIV).

^b^ Abbreviations: N = total number of participants, n(%) = number (percentage) of participants with characteristic, min–max = minimum to maximum, SD = standard deviation.

### Immunogenicity

#### HI response

**Figure 2:**
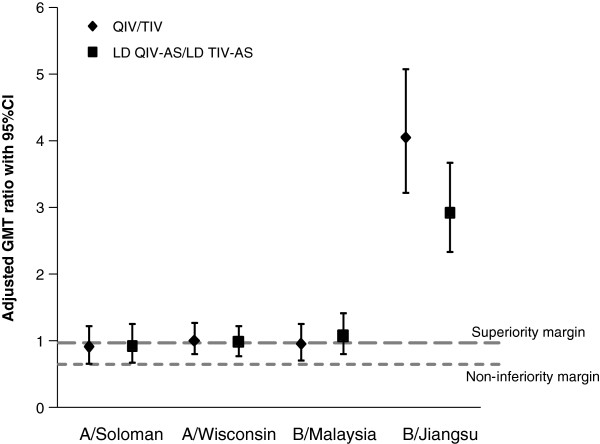
**Adjusted GMT ratios on Day 21 (ATP cohort for immunogenicity).** Adjusted GMT ratios for A/Solomon Islands, A/Wisconsin, B/Malaysia and B/Jiangsu (only included in QIV formulations) on Day 21 for all treatment groups in the per-protocol cohort for immunogenicity. The margin for superiority of response was if the 95% CI lower limit was ≥1 and the margin for non-inferiority of response was if the 95% CI lower limit was ≥0.67. The data points represent the adjusted GMT ratio for QIV group over the TIV group (QIV/TIV; diamond) or the LD QIV-AS group over the LD TIV-AS group (LD QIV-AS/LD TIV-AS; square) with their associated 95% confidence intervals (CIs). Abbreviations: LD QIV-AS: low-dose adjuvanted quadrivalent influenza vaccine; LD TIV-AS: low-dose adjuvanted trivalent influenza vaccine; QIV: quadrivalent influenza vaccine; TIV: trivalent influenza vaccine.

All four study vaccines exceeded both the CHMP criteria set for the yearly evaluation of seasonal influenza vaccines in participants aged 18 to 60 years [25] and the CBER criteria for the licensure of influenza vaccines in adults aged 18 to 64 years [24] for the three recommended seasonal strains (A/Solomon Islands, A/Wisconsin and B/Malaysia strains). Only the QIV and LD QIV-AS vaccines met all the CHMP and the CBER criteria for the B/Jiangsu strain (Table 2). QIV and LD QIV-AS were shown to be non-inferior to TIV and LD TIV-AS for the three strains contained in the TIV; the lower limits of the 95% CI of GMT ratios for HI antibodies to the three recommended seasonal strains were above the pre-defined limit of 0.67 (Figure 2). Superiority of the response elicited by both QIV and LD QIV-AS as compared to TIV and LD TIV-AS was also shown for the B/Jiangsu strain. Lower limits of the 95% CI of GMT ratios for HI antibodies to the B/Jiangsu strain were above the pre-defined limit of 1 (Figure 2), with responses 3 to 4 times higher with the QIV formulations compared to the TIV vaccines. Furthermore, the immunogenicity of LD QIV-AS was shown to be non-inferior to that of QIV with the adjusted GMT ratios (LD QIV-AS/QIV) being 1.15 (95% CI 0.85–1.55) for A/Solomon Islands, 1.21 (0.97–1.52) for A/Wisconsin, 1.16 (0.88–1.53) for B/Malaysia and 0.89 (0.71–1.11) for B/Jiangsu.

**Table 2:** Summary of immunogenicity for the per-protocol cohort

**Treatment group**^**a**^					
**Vaccine Strain**		**QIV**	**LD QIV-AS**	**LD TIV-AS**	**TIV**
N^b^		104	104	104	105
A/Solomon Islands					
GMT (95% CI)	Pre	21.4 (16.1–28.4)	22.2 (16.6–29.5)	23.3 (17.5–31.0)	18.4 (14.3–23.7)
	Post	130.0 (106.1–159.4)	150.6 (118.4–191.5)	160.4 (129.1–199.3)	133.8 (105.6–169.7)
SCR %(95% CI)	Post	56.7 (46.7–66.4)*^$^	57.7 (47.6–67.3)* ^$^	54.8 (44.7–64.6)* ^$^	60.0 (50.0–69.4)* ^$^
SPR %(95% CI)	Pre	37.5 (28.2–47.5)	42.3 (32.7–52.4)	40.4 (30.9–50.5)	35.2 (26.2–45.2)
	Post	92.3 (85.4–96.6)* ^$^	88.5 (80.7–93.9)* ^$^	93.3 (86.6–97.3)* ^$^	90.5 (83.2–95.3)* ^$^
SCF (95% CI)	Post	6.1 (4.6–8.0)*	6.8 (5.0–9.2)*	6.9 (5.0–9.4)*	7.3 (5.3–9.9)*
A/Wisconsin					
GMT (95% CI)	Pre	29.3 (23.0–37.3)	25.7 (19.8–33.3)	30.7 (23.7–39.8)	29.0 (22.5–37.4)
	Post	162.1 (138.0–190.4)	189.5 (158.9–226.0)	197.9 (169.1–231.7)	156.3 (127.5–191.6)
SCR %(95% CI)	Post	60.6 (50.5–70.0)* ^$^	66.3 (56.4–75.3)* ^$^	64.4 (54.4–73.6)* ^$^	59.0 (49.0–68.5)* ^$^
SPR %(95% CI)	Pre	51.0 (41.0–60.9)	46.2 (36.3–56.2)	53.8 (43.8–63.7)	55.2 (45.2–65.0)
	Post	97.1 (91.8–99.4)* ^$^	98.1 (93.2–99.8)* ^$^	100 (96.5–100)* ^$^	96.2 (90.5–99.0)* ^$^
SCF (95% CI)	Post	5.5 (4.4–6.9)*	7.4 (5.8–9.4)*	6.4 (5.0–8.3)*	5.4 (4.1–7.0)*
B/Malaysia					
GMT (95% CI)	Pre	32.2 (24.8–41.8)	26.6 (20.2–35.0)	23.2 (17.7–30.4)	27.2 (20.6–35.8)
	Post	192.8 (159.6–232.9)	213.0 (174.0–260.9)	187.0 (151.9–230.3)	188.5 (150.0–237.0)
SCR %(95% CI)	Post	57.7 (47.6–67.3)* ^$^	65.4 (55.4–74.4)* ^$^	56.7 (46.7–66.4)* ^$^	59.0 (49.0–68.5)* ^$^
SPR %(95% CI)	Pre	51.0 (41.0–60.9)	47.1 (37.2–57.2)	42.3 (32.7–52.4)	44.8 (35.0–54.8)
	Post	97.1 (91.8–99.4)* ^$^	97.1 (91.8–99.4)* ^$^	96.2 (90.4–98.9)* ^$^	93.3 (86.7–97.3)* ^$^
SCF (95% CI)	Post	6.0 (4.7–7.7)*	8.0 (6.1–10.5)*	8.1 (5.9–11.0)*	6.9 (5.2–9.3)*
B/Jiangsu					
GMT (95% CI)	Pre	19.6 (15.6–24.8)	19.5 (15.4–24.5)	23.4 (18.3–29.7)	19.2 (15.2–24.3)
	Post	179.1 (151.4–211.9)	158.3 (130.7–191.9)	59.2 (47.3–74.0)	43.4 (34.5–54.6)
SCR %(95% CI)	Post	76.0 (66.6–83.8)* ^$^	78.8 (69.7–86.2)* ^$^	26.9 (18.7–36.5)	19.0 (12.0–27.9)
SPR %(95% CI)	Pre	31.7 (22.9–41.6)	38.5 (29.1–48.5)	43.3 (33.6–53.3)	39.0 (29.7–49.1)
	Post	98.1 (93.2–99.8)* ^$^	95.2 (89.1–98.4)* ^$^	75.0 (65.6–83.0)*	63.8 (53.9–73.0)
SCF (95% CI)	Post	9.1 (7.2–11.5)*	8.1 (6.6–10.0)*	2.5 (2.1–3.0)*	2.3 (1.9–2.6)

^a^ Groups were administered the quadrivalent influenza vaccine (QIV), the low-dose adjuvanted quadrivalent influenza vaccine (LD QIV-AS), the low-dose adjuvanted trivalent influenza vaccine (LD TIV-AS) or the trivalent influenza vaccine (TIV).

^b^ Abbreviations: 95% CI = 95% confidence interval (lower limit–upper limit); GMT = geometric mean titer; N = total number of participants; Pre = Pre-vaccination (Day 0); Post = Post-vaccination (Day 21); SCR = Seroconversion ratio defined as percentage of participants with antibody titer ≥1:40 1/DIL post-vaccination for initially seronegative participants, or ≥4 fold the pre-vaccination antibody titer for initially seropositive participants; SPR = Seroprotection rate defined as percentage of participants with antibody titer ≥1:40; SCF = Seroconversion factor defined as the fold increase in GMTs post-vaccination compared with pre-vaccination.

* CHMP criteria were met or exceeded (SCR > 40%, SPR > 70%, SCF > 2.5).

^$^ CBER criteria were met or exceeded (95% CI lower limit for SCR ≥ 40%, SPR ≥ 70%).

#### MN response

**Figure 3:**
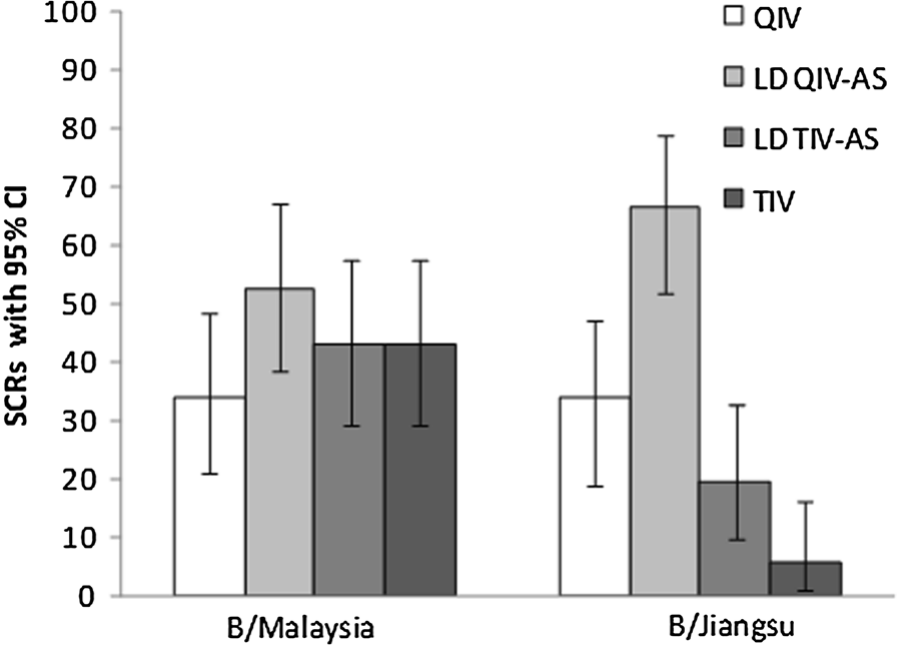
**Summary of results of microneutralizing antibody assay for B influenza strains.** Note: Seroconversion rates (SCRs) of the microneutralizing antibody assay in all treatment groups. Neutralizing antibody responses to B/Malaysia and B/Jiangsu were measured before vaccination (Day 0, Pre) and 21 days post-vaccination (Day 21, Post). SCRs, defined as ≥4-fold increase in titer relative to the value noted at baseline, are presented with the associated 95% CI. Abbreviations: LD QIV-AS: low-dose adjuvanted quadrivalent influenza vaccine; LD TIV-AS: low-dose adjuvanted trivalent influenza vaccine; QIV: quadrivalent influenza vaccine; TIV: trivalent influenza vaccine.

On Day 21, the GMTs (95% CI) for the neutralizing antibodies to B/Malaysia strain were: 226.1 [165.7–308.6] in QIV group, 316.1 [226.3–441.6] in LD QIV-AS group, 296.9 [206.2–427.4] in LD TIV-AS group and 255.8 [183.8–355.9] in TIV group, with the CIs overlapping between the trivalent and the quadrivalent formulations. For the B/Jiangsu strain the highest CIs were observed for the quadrivalent vaccines (QIV: 157.0 [115.5–213.4], LD QIV-AS: 161.1 [120.9–214.7], LD TIV-AS 65.6 [47.9–89.9] and TIV: 38.4 [29.3–50.3]). Similar observations were made for SCR on Day 21 (Figure 3).

### Safety

The majority of participants (range: 67.6%–86.5%) reported at least one symptom (solicited or unsolicited) during the 7-day post-vaccination period (Table 3). Local symptoms were reported more often than general symptoms for all vaccine groups. Grade 3 symptoms (solicited and unsolicited) were reported by 3.8% (TIV), 3.8% (QIV), 9.5% (LD TIV-AS) and 4.8% (LD QIV-AS) of participants. Pain at the injection site was the most frequently reported solicited local symptom for all vaccine groups (range: 49.5%–76.0%, Table 3). Grade 3 pain occurrence was low in the adjuvanted groups (LD QIV-AS: 1.9% and LD TIV-AS: 2.9%), and absent with the unadjuvanted vaccines. The incidence of other solicited local symptoms (redness and swelling at the injection site) was low (≤ 6.7%, Table 3). Fatigue (range: 30.5%–45.2%), myalgia (14.3%–38.5%) and headache (21.9%–31.7%) were the most frequently reported solicited general symptoms considered to be related to vaccination for all vaccine groups. Grade 3 general reactogenicity was low (≤ 7.6%, Table 3). One SAE was reported in the TIV Group (prolonged hospitalization for hemorrhage after tonsillectomy), which was not considered to be related to vaccination. No fatal SAEs occurred and no pIMD were reported up to day 180 after vaccination.

**Table 3:** Safety and reactogenicity on Days 0–6 post-vaccination (TVC)

**Treatment group**^**a**^				
**Symptom % (95% CI)**^**b**^	**QIV**	**LD QIV-AS**	**LD TIV-AS**	**TIV**
N^c^	105	104	105	105
Any symptom	79.0 (70.0–86.4)	86.5 (78.4–92.4)	77.1 (67.9–84.8)	67.6 (57.8–76.4)
Grade 3^d^	3.8 (1.0–9.5)	4.8 (1.6–10.9)	9.5 (4.7–16.8)	1.9 (0.2–6.7)
Local Symptoms	72.4 (62.8–80.7)	76.9 (67.6–84.6)	70.5 (60.8–79.0)	49.5 (39.6–59.5)
Grade 3	0 (0–3.5)	1.9 (0.2–6.8)	2.9 (0.6–8.1)	0 (0–3.5)
Pain	72.4 (62.8–80.7)	76.0 (66.6–83.8)	70.5 (60.8–79.0)	49.5 (39.6–59.5)
Grade 3	0 (0–3.5)	1.9 (0.2–6.8)	2.9 (0.6–8.1)	0 (0–3.5)
Redness	2.9 (0.6–8.1)	5.8 (2.1–12.1)	4.8 (1.6–10.8)	1.0 (0–5.2)
>100 mm	0 (0–3.5)	0 (0–3.5)	0 (0–3.5)	0 (0–3.5)
Swelling	2.9 (0.6–8.1)	3.8 (1.1–9.6)	6.7 (2.7–13.3)	1.9 (0.2–6.7)
>100 mm	0 (0–3.5)	0 (0–3.5)	0 (0–3.5)	0 (0–3.5)
General Symptoms	44.8 (35.0–54.8)	57.7 (47.6–67.3)	53.3 (43.3–63.1)	43.8 (34.1–53.8)
Grade 3	3.8 (1.0–9.5)	4.8 (1.6–10.9)	7.6 (3.3–14.5)	1.9 (0.2–6.7)
Arthalgia	5.7 (2.1–12.0)	24.0 (16.2–33.4)	12.4 (6.8–20.2)	10.5 (5.3–18.0)
Grade 3	1.0 (0–5.2)	2.9 (0.6–8.2)	1.9 (0.2–6.7)	0 (0–3.5)
Fatigue	30.5 (21.9–40.2)	45.2 (35.4–55.3)	34.3 (25.3–44.2)	31.4 (22.7–41.2)
Grade 3	1.9 (0.2–6.7)	3.8 (1.1–9.6)	2.9 (0.6–8.1)	1.0 (0–5.2)
Headache	22.9 (15.2–32.1)	31.7 (22.9–41.6)	24.8 (16.9–34.1)	21.9 (14.4–31.0)
Grade 3	2.9 (0.6–8.1)	1.9 (0.2–6.8)	1.0 (0–5.2)	0 (0–3.5)
Myalgia	16.2 (9.7–24.7)	38.5 (29.1–48.5)	31.4 (22.7–41.2)	14.3 (8.2–22.5)
Grade 3	1.0 (0–5.2)	2.9 (0.6–8.2)	2.9 (0.6–8.1)	1.0 (0–5.2)
Nausea	7.6 (3.3–14.5)	9.6 (4.7–17.0)	7.6 (3.3–14.5)	7.6 (3.3–14.5)
Grade 3	1.9 (0.2–6.7)	1.9 (0.2–6.8)	1.9 (0.2–6.7)	1.0 (0–5.2)
Shivering	3.8 (1.0–9.5)	9.6 (4.7–17.0)	8.6 (4.0–15.6)	3.8 (1.0–9.5)
Grade 3	1.0 (0–5.2)	1.9 (0.2–6.8)	1.0 (0–5.2)	0 (0–3.5)
Fever (°C)	1.0 (0–5.2)	2.9 (0.6–8.2)	1.9 (0.2–6.7)	1.0 (0–5.2)
>39°C	0 (0–3.5)	0 (0–3.5)	0 (0–3.5)	0 (0–3.5)

^a^ Groups were administered the quadrivalent influenza vaccine (QIV), the low-dose adjuvanted quadrivalent influenza vaccine (LD QIV-AS), the low-dose adjuvanted trivalent influenza vaccine (LD TIV-AS) or the trivalent influenza vaccine (TIV).

^b^ Abbreviations: 95% CI = 95% confidence interval (lower limit–upper limit).

^c^ N = total number of participants with symptom sheets returned.

^d^ Grade 3 symptoms were defined as symptoms that prevented normal everyday activity.

Table displays local and general symptoms reported as related to vaccination. All reported local symptoms were considered related to vaccination.

## Discussion

Trivalent influenza vaccines recommended for immunization against influenza contain the two A-strains (H1N1 and H3N2) but only one B-strain selected each year in the Yamagata-like lineage group or the Victoria-like one [9,29]. The efficacy and effectiveness of influenza vaccines depend on the degree of similarity between the circulating viruses and those included in the vaccine [12].

The present study was conducted to examine how the addition of another strain would affect the immune response and reactogenicity observed with a TIV. The addition of a fourth strain in the QIV vaccine formulations did not impact the immune response elicited in healthy adults aged 18 to 60 years to the three strains contained in the TIV vaccines, as demonstrated by the statistical non-inferiority of the immunological response elicited by QIV and LD QIV-AS over TIV and LD TIV-AS 21 days after vaccination. Furthermore, the response elicited against the B/Jiangsu strain contained in the QIV formulations, but not in the trivalent vaccines was shown to be superior in terms of levels of antibodies reached 21 days after vaccination. As shown in other studies [30], some levels of cross reactive antibodies were elicited in this adult population against the heterologuous B strains by the TIV formulations. However, levels of antibodies indicative of seroprotection were only reached with the QIV formulations in the present study. The criteria as defined in the CHMP and CBER guidelines for seasonal influenza vaccines in adults were met for the A/Solomon Islands, A/Wisconsin and B/Malaysia strains for all four vaccine groups. Nevertheless, only the quadrivalent formulations were able to elicit the level of immunological response required to fulfill all CHMP and CBER criteria against the B/Jiangsu strain 21 days after vaccination. The micro-neutralization assay results confirmed the data obtained with HI assay. Similar results were previously reported with a quadrivalent live attenuated vaccine containing two influenza subtype A strains (A/H1N1 and A/H3N2) and two B strains (B/Yamagata and B/Victoria). This live attenuated vaccine was shown to be non-inferior to trivalent live attenuated vaccines both in adults and in children [31,32].

As a clinical impact on vaccine efficacy has been suggested or described in several studies when the circulating strain belonged to the other B lineage [13,14,30,33-35], the immunological improvement elicited by QIV formulations is likely to translate into a direct clinical benefit. Additionally, the use of quadrivalent vaccines including an influenza B strain representing both lineages could result in substantial cost-savings. Recently, two modeling papers [36,37] evaluated the potential benefits associated with the use of a quadrivalent vaccine compared with a TIV for the time period 1999–2009 in the US. Based on the clinical burden of influenza illness and viral surveillance data, the Center for Disease Control and Prevention estimated that the use of a QIV rather than a TIV between 1999 and 2009 could have resulted in additional reductions in the number of cases of influenza illnesses, influenza-associated respiratory hospitalizations, and influenza-related respiratory deaths [36]. Furthermore, Lee *et al.* calculated that the use of QIV during the same time period may have been associated with substantial cost-savings for society and third party payers (median savings: $3.1 billion and $292 million, respectively) [37].

Another objective of the present study was to see whether an adjuvant would trigger cross reactive antibodies against the 2 B lineages. GlaxoSmithKline Vaccines proprietary α-tocopherol, oil-in-water emulsion-based Adjuvant System, AS03, has been shown to elicit cross-reactive response against drifted influenza strains [21]. In the present study adjuvant alone was not sufficient to induce a cross-reactive immune response to the B/Jiangsu strain, which may be expected as the two B-strains are genetically too distinct [3] and so may not be simply considered as drift variants. However, a dose-sparing effect has been demonstrated with the use of 5 μg HA per strain in the adjuvanted formulations (20 μg HA/dose) instead of 15 μg in the non-adjuvanted vaccines (60 μg HA/dose), as illustrated by the immunological non-inferiority of LD QIV-AS vs. QIV. This is in line with other studies using AS03 adjuvant [21,38].

In terms of reactogenicity, no clinically relevant impact was found with the addition of a fourth strain in the vaccines. The safety profile of the study vaccines was comparable to that seen in other studies conducted in adults [39-41]. The non adjuvanted QIV formulation reactogenicity profile was generally similar to the TIV vaccine. As expected, the adjuvanted vaccines had a higher reactogenicity profile compared to the non-adjuvanted formulations, being associated with a higher incidence of solicited injection site and general symptoms. Similar observations were reported in previous clinical trials of influenza A/H1N1pdm09 [38,42-44] and H5N1 [45] vaccines comparing safety outcomes between adjuvanted and non-adjuvanted vaccines. Local and general adverse events tended to be more frequent with adjuvanted vaccines compared with non-adjuvanted vaccines, in both adults and children. No potentially related serious adverse events were reported during the conduct of the study. However, our study with 105 vaccines per vaccine group was not designed to assess rare events.

Another limitation of the current study is the absence of further follow-up of the participants. Additional information (beyond 21 days post vaccination) on the immune responses to the additional B-strain, including data on the persistence of antibodies against B/Jiangsu strain during the influenza season, would be of interest. Another limitation is that the study enrolled relatively young participants (<60 years of age). Whether our findings can be extended to other populations such as the elderly, with decreased immune response to influenza vaccines [46] remains to be evaluated.

## Conclusions

As observed by others [1,2], this study confirms that adults vaccinated against one B-lineage type have reduced post-vaccination GMTs and a lower percentage of titers ≥40 to viruses from the alternative lineage. Consequently, adults vaccinated with influenza vaccine against one type B-lineage would have reduced protection against infection with the heterologuous lineage if it were circulating. The use of a quadrivalent influenza vaccine, containing B-strains from both circulating lineages, could therefore provide a broader protection against influenza infection, while maintaining a similar safety profile.

This study, conducted at a single site in Czech Republic, provides evidence of the benefit of QIV vaccination to the participants of this study aged between 18 and 60 years. Other clinical studies have been conducted to confirm this finding in a broader adult population, as well as in other populations, including children and elderly persons (registered at http://www.ClinicalTrials.gov: NCT01204671 and NCT01196988).

## Competing interests

Mathieu Peeters, Walthère Dewé and Jeanne-Marie Devaster are employees of GlaxoSmithKline group of companies and report ownership of stock options. Walthère Dewé also declares having received funding to travel, accommodations, or meeting expenses from GlaxoSmithKline group of companies. Jiri Beran declares his institution received a grant to conduct the present clinical trial and support for travel to meeting for study presentation from GlaxoSmithKline group of companies. Lenka Hobzova and Jolana Raupachova declare their institution received a grant to conduct the present clinical trial from GlaxoSmithKline group of companies.

## Authors’ contributions

All authors participated in the design, implementation, analysis and/or interpretation of the study. Mathieu Peeters and Jeanne-Marie Devaster led the clinical team at GlaxoSmithKline group of companies and were involved in all phases of the study. Jiri Beran coordinated the study at the investigator site. Jolana Raupachova and Lenka Hobzova contributed as site investigators. Walthère Dewé conducted the statistical analysis. All the authors revised the manuscript critically for important intellectual content and approved the final version before submission.

## Pre-publication history

The pre-publication history for this paper can be accessed here:

http://www.biomedcentral.com/1471-2334/13/224/prepub
